# A Letter from Our Arkansas Correspondent

**Published:** 1888-06

**Authors:** H. S. Robertson

**Affiliations:** Peytonville, Arkansas


### LETTER FROM OUR ARKANSAS CORRESPONDENT.

### CONGENITAL OCCLUSION OF THE ANUS.

*Editor Daniel's Texas Medical Journal:*

There is hardly any one whose experience is so limited that now and then a rare case does not occur. It was the writer's fortune, on April 30, to be called in consultation with Dr. C., of New Hope, Arkansas, the subject being an infant, in a well-to-do and very respectable negro family. Upon arriving, we found what we at once diagnosed occluded anus. The case having existed for two or three days, under the treatment of an old midwife for colic, the contents of the rectum had made its way through the substance of the gut, and into the scrotal sack, from whence it escaped by a pen point opening in the median line of the scrotum, and just below the penis, consequently the scrotum was very much distended with liquid fecus. We immediately begun to operate, in the simplest manner possible, by first slightly enlarging the extended scrotal opening, until it would admit a small sized probe, which we introduced, passing through the sack and into the bowel below, then by carrying the external portion of the probe well up towards the abdomen, the end within the rectum could be easily felt a little above and to the left of where the anus should have been. With a sharp-pointed knife, we cut down upon this end of the probe, finishing the operation with a probe pointed bistoury, the immediate result of which was a copious discharge of very foul smelling excretia, and instant relief to the child. I do not report this for the purpose of extolling it as a great surgical exploit, nor to advocate any new plan of procedure for which I expect to obtain a patent, but simply to show that such cases do occur, even in the practice of the country doctor, that this class of practitioners, who may read this, will not be caught unsuspecting, as I was.

At this writing, six days since the operation, the child is doing well, with apparently as good prospects for a long life as any child.

H. S. Robertson, M. D.,
Peytonville, Arkansas.

